# Characterization and Management of Adverse Events of Low-Dose Oral Minoxidil Treatment for Alopecia: A Narrative Review

**DOI:** 10.3390/jcm14061805

**Published:** 2025-03-07

**Authors:** Juan Jimenez-Cauhe, Kristen I. Lo Sicco, Jerry Shapiro, Angela Hermosa-Gelbard, Patricia Burgos-Blasco, Ana Melian-Olivera, Daniel Ortega-Quijano, Cristina Pindado-Ortega, Diego Buendia-Castaño, Daniel Asz-Sigall, Sergio Vaño-Galvan

**Affiliations:** 1 Department of Dermatology, Hospital Universitario Ramon y Cajal, IRYCIS, Universidad de Alcala, 28034 Madrid, Spain; 2 Hair Disorder Unit, Grupo Pedro Jaén, 28002 Madrid, Spain; 3 The Ronald O Perelman Department of Dermatology, NYU Langone Health, New York, NY 10016, USA; 4 Trichology Clinic, Hospital General Dr Manuel Gea Gonzalez, Mexico City 14080, Mexico

**Keywords:** alopecia, hair loss, oral minoxidil, tachycardia, arrhythmia, edema, hypertrichosis

## Abstract

Low-dose oral minoxidil (LDOM) has emerged as a widely used off-label treatment for different types of alopecia, showing a favorable safety profile and effectiveness. Despite its growing use, it is essential to understand the possible associated adverse events (AEs) and their appropriate management to optimize this therapy. The aim of this article was to comprehensively review the AEs of LDOM treatment, describing their frequency, risk factors, affected anatomical sites, and management strategies. A search in the PubMed and EMBASE databases was performed for studies published before 31 December 2024, reporting the treatment of any type of hair loss with oral minoxidil. The most frequent AE is hypertrichosis, occurring in approximately 15% of patients, with a higher incidence in women and patients with higher doses. Fluid retention affects 1.3–10% of patients, particularly women, and typically occurs within 1–3 months of treatment. Other cardiovascular AEs, such as tachycardia or dizziness, occur in fewer than 5% of cases and are usually mild and transient. Severe AEs, including pericardial effusion, are extremely rare and often linked to compounding errors comprising an excessive dose. Management strategies include dose reduction, pharmacological interventions like diuretics for edema, and lifestyle measures such as sodium restriction. In most cases, AEs resolve without the need for treatment discontinuation. The favorable safety profile of LDOM makes it a valuable therapeutic option for alopecia, though careful patient selection, dose titration, and monitoring are essential to minimize risks.

## 1. Introduction

Oral minoxidil is a drug only approved by the FDA for the treatment of severe refractory hypertension [1]. However, its use as an antihypertensive has declined with the emergence of newer, more effective, and better-tolerated drug classes for this indication. Antihypertensive doses typically range from 10 to 40 mg daily, escalating to 100 mg in refractory cases. At these doses, numerous adverse effects (AEs) have been reported, which initially limited its use for hair loss by dermatologists [1]. In recent years, low-dose oral minoxidil (LDOM) has evolved from an emerging therapy to one of the most widely used treatments for different types of alopecia (including androgenetic alopecia, telogen effluvium, chemotherapy-induced alopecia, frontal fibrosing alopecia or lichen plano pilaris), with numerous studies supporting its safety and effectiveness [2,3,4,5]. In fact, LDOM has recently been included as a first-line therapy for androgenetic alopecia (AGA) in both women and men in the guidelines of the Spanish Trichology Group, with usual doses ranging from 0.5 to 1 mg and 2.5 to 5 mg daily, respectively [6]. It is recommended to start with initial doses (0.5 or 0.625 mg daily in women and 2.5 daily in men) and up-titrate according to tolerability and clinical response [2,6].

**Figure 1:**
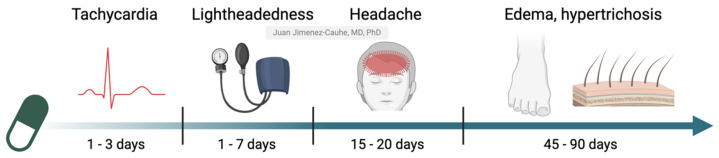
Chronological pattern of onset for the main adverse effects of LDOM. Adapted with permission from the work of Vaño-Galvan et al., 2021 [2]. Created with Biorender.com.

However, it is important to remember that LDOM is still an off-label treatment, and patient safety must remain a priority. Therefore, it is essential for dermatologists to know and manage its possible AEs. This article aims to provide a comprehensive review of the AEs associated with LDOM, describing their frequency, chronology, clinical characteristics, and management strategies (Figure 1). By addressing these issues, the goal is to optimize the safety and effectiveness of the treatment while facilitating informed clinical decision-making.

## 2. Methods

A search in the PubMed and EMBASE databases was performed using our institutional electronic library to identify studies reporting the use of oral minoxidil in the treatment of any type of hair loss. The search included articles published before 31 December 2024 that contained in the title the terms “minoxidil” and “oral” or “orally” or “systemic” (minoxidil[Title]) AND (oral[Title] OR orally[Title] OR systemic[Title]). All types of studies were contemplated for this narrative review, including randomized clinical trials, prospective and retrospective observational studies, case series, case reports, systematic or narrative reviews and guidelines of scientific societies. After duplicate studies were removed, the articles were screened and excluded according to the objectives of this review. Studies that focused on oral minoxidil pharmacology, in vitro or in vivo animal models, or other applications rather than hair diseases were excluded. In an effort to include all available studies about this topic, other studies were identified after reviewing the relevant articles’ references.

## 3. Hypertrichosis

### 3.1. Frequency and Risk Factors

Hypertrichosis is the most common AE during treatment with LDOM, with a frequency ranging between 4 and 93% of the patients, according to different series [7,8]. The largest study by Vaño-Galvan et al. found a frequency of 15.1% [2]. This widely variable incidence may be explained by different factors influencing the development of hypertrichosis:

- ***Minoxidil dose.*** Dose is the main factor associated with hypertrichosis, with a statistically significant association [2,9,10]. A meta-analysis of individual patient data found that the frequency of hypertrichosis increased with higher doses of LDOM [10]. Other recent meta-analyses have found similar results, confirming that hypertrichosis is a dose-dependent AE [9,11]. It is estimated that for every up-titration of 1 mg, the risk of hypertrichosis increases by 17.6% [12].
- ***Gender and age.*** Several studies have shown a higher frequency of hypertrichosis in women than in men [2,3,9]. This may seem contrary to the aforementioned dose-dependent relationship, given that men usually receive higher doses than women. However, there is probably a reporting bias in the detection of this AE since many men do not perceive the increase in body and facial hair as an AE or are not even aware of it, while, in our experience, women are usually more concerned about this AE. In this sense, it has been observed that the average dose to produce hypertrichosis is higher in men (4.1 mg) than in women (1.4 mg) [2]. In addition, dose reduction or discontinuation of LDOM due to hypertrichosis is much more common in women than in men [13]. In terms of age, only one study found that younger age was associated with an increased risk of hypertrichosis in men [4].
- ***Phototype.*** Although it has not been objectively studied, patients with dark-colored hair tend to have more obvious hypertrichosis than those with light hair [3]. In fact, it can be observed that the reported rate of hypertrichosis is usually higher in studies carried out in geographical areas where dark-colored hair predominates, such as Brazil or Thailand [4,8].
- ***Pharmaceutical form and posology.*** Vaño-Galvan’s study showed an increased risk of hypertrichosis in patients taking minoxidil compounded capsules compared to those taking commercially available tablets, suggesting that it may be due to some dose variability in the compounded dosages. Additionally, an every-other-day regimen was associated with a lower risk of hypertrichosis compared to a daily regimen; however, this observation was not substantiated with a plausible explanation [2].
- ***Concomitant treatments.*** Several studies suggest that concomitant treatment of LDOM with androgen receptor inhibitor drugs, such as bicalutamide or spironolactone, may reduce the frequency of hypertrichosis.

Moussa et al. described an improvement in LDOM-induced facial hypertrichosis in 35 women after initiating treatment with bicalutamide. The mean dose of bicalutamide that achieved an improvement in hypertrichosis was 14.4 mg daily, and the median time to improvement was 3.4 months. This allowed for an increase in the mean dose of minoxidil from 1.5 to 2.2 mg without producing hypertrichosis [14]. This article and its results have been questioned, and there is controversy about the preventive effect of bicalutamide on hypertrichosis [9,15].

Regarding spironolactone, Olamiju et al. conducted a study in six female adolescents (aged 13 to 18) with AGA treated with LDOM 2.5 mg and spironolactone 50–100 mg. They found that no patient developed hypertrichosis, attributing this to a possible beneficial effect of spironolactone [16]. A previous study in 12 women with a mean age of 7 years diagnosed of “idiopathic hypertrichosis” reported a reduction in hypertrichosis with spironolactone 50–100 mg, with subsequent recurrence upon discontinuation [17]. A recent study by Nohria et al. evaluated hypertrichosis in 54 female patients. Although not statistically significant, it showed that the group treated with LDOM (mean dose 1.5 mg) plus spironolactone (mean dose 104 mg) had a lower frequency of hypertrichosis compared to those treated with LDOM in monotherapy (17.6% vs. 40.5%; *p* = 0.13) [18].

### 3.2. Affected Areas

**Figure 2:**
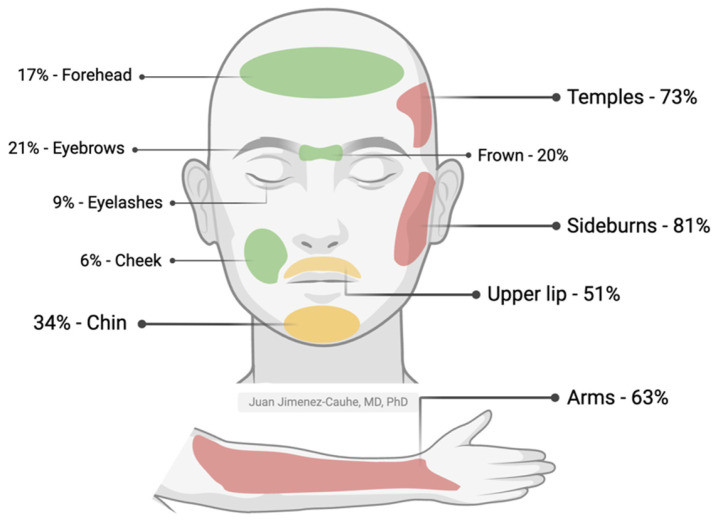
Frequency of LDOM-induced hypertrichosis in facial and body areas. The colors represent a high (red), medium (yellow), or low (green) frequency of hypertrichosis. Adapted with permission from the work of Jimenez-Cauhe et al., 2021 [13]. Created with Biorender.com.

The appearance of unwanted hair usually occurs on the face or extremities and, less commonly, on the trunk. In patients who develop LDOM-induced hypertrichosis, the most frequently affected areas are the temples and sideburns, followed by arms, upper lip (mustache), and chin. Other less common areas include between the eyebrows, forehead, cheeks, and dorsum of hands, followed by the legs and trunk (Figure 2) [2,13,14,19]. A Brazilian study of 435 patients showed that 68.9% of patients with hypertrichosis had two or more affected areas [4].

In men, an increase in beard hair has been reported in up to 52% of patients [7]. Interestingly, this may be a positive effect in many cases rather than an AE. In fact, topical minoxidil has also been used to increase facial hair in cis men and trans men [20,21].

Another special location is the eyebrows and eyelashes. Increase in eyebrow hair has been reported in up to 21% of patients treated with LDOM [4]. In some cases, especially in patients with frontal fibrosing alopecia (FFA), it can be perceived as a positive effect [22]. In this regard, Pirmez et al. reported cosmetically acceptable eyebrow regrowth in seven women with FFA treated with LDOM 1.25–2.5 mg [23]. Clinical experience suggests that eyelashes usually experience growth during LDOM treatment; however, studies are necessary to fully evaluate this benefit. Some articles estimate a lower frequency than in other locations in up to 9% of patients [4,22].

### 3.3. Severity and Management

**Figure 3:**
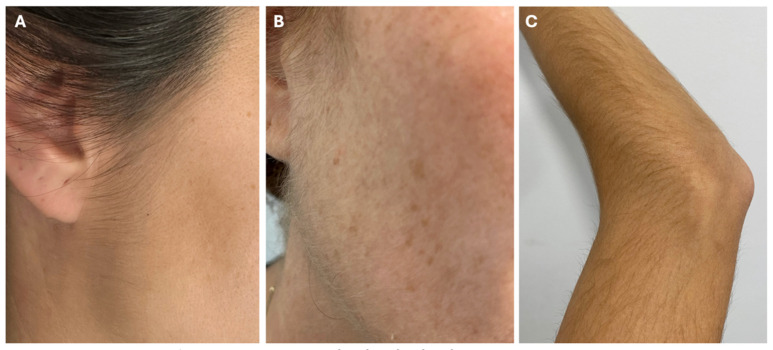
Varying degrees of LDOM-induced hypertrichosis and their management. (**A**) Typical mild hypertrichosis on sideburns, not concerning for the patient. (**B**) Moderate hypertrichosis on sideburns, cheeks and neck, treated with bleaching. (**C**) Moderate hypertrichosis on arms, which required dose reduction due to patient concern regarding this AE.

Despite being the most common AE, hypertrichosis is usually mild, and rarely requires discontinuation of LDOM [2,13]. However, in our experience, it is the main reason for women to decide against dose escalation, which might impact the effectiveness of LDOM at lower doses. In Vaño-Galvan’s study of 1404 patients, only 0.5% of patients discontinued LDOM due to hypertrichosis, while in Sanabria’s study of 435 patients, the discontinuation rate was 1.6% [2,4]. Most patients are not concerned with or prefer to remove the unwanted hair rather than stop the treatment (Figure 3) [13]. The most preferred options are laser or intense pulsed light (IPL), which offer more permanent hair removal [13]. Other temporary techniques include bleaching, waxing, shaving, threading, sugaring, and dermaplaning [13,24]. Another less common alternative for facial hair is the use of topical eflornithine, a drug approved to treat facial hirsutism [11,25]. In more severe or worrisome cases of hypertrichosis, an LDOM dose adjustment is necessary. This occurs in approximately 4% of patients treated with LDOM, and in most of them, hypertrichosis resolves with dose reduction [2,13].

## 4. Blood Pressure

### 4.1. Influence of LDOM on Blood Pressure

Minoxidil is a direct arteriolar vasodilator that decreases blood pressure by opening the ATP-dependent potassium channels of vascular smooth muscle cells. This results in hyperpolarization of the cell membrane, preventing the entry of calcium ions, resulting in smooth muscle relaxation and vasodilation [26]. However, in contrast to oral minoxidil at standard doses for hypertension (10–40 mg per day), LDOM has shown to have little or no influence on blood pressure (BP) in normotensive patients. A study from 1989 assessed the pharmacokinetics of oral minoxidil at daily doses of 2.5, 5 and 10 mg in 29 healthy volunteers, showing a slight reduction in BP, with no significant differences between the three doses [27]. In recent years, new studies have evaluated BP, either after the first dose of LDOM, or after 24 weeks of treatment. Overall, they showed either a non-significant reduction in BP, or a statistically significant but not clinically relevant reduction (Table 1) [8,28,29,30,31,32]. This has been supported by a recent meta-analysis that included 2387 patients from 16 studies. No statistically significant variation was observed in systolic BP (SBP), diastolic BP (DBP) or mean arterial pressure (MAP) [33]. Among them, three studies performed ambulatory blood pressure monitoring (ABPM) for 24 h [30,31,32].

Sanabria et al. performed 24 h ABPM in 34 patients at baseline and at 24 weeks of treatment with minoxidil 5 mg, finding a statistically significant reduction in MAP, from 92.6 to 89.8 mmHg (*p* = 0.015). However, this reduction was clinically irrelevant, and none of the patients had clinical hypotension [30]. Recently, these authors conducted a study in a subgroup of 11 male patients (mean age 37.9 years) who escalated the LDOM dose from 5 mg to 7.5 mg daily. Two 24 h ABPM measures were performed at baseline (LDOM 5 mg) and at 6 weeks of treatment with LDOM 7.5 mg, finding no significant changes in BP. They did find a significant increase in heart rate (HR) of 5.6 bpm. No cardiovascular AEs were detected [34].

Jimenez-Cauhe et al. carried out a similar study in 10 male patients who started treatment with minoxidil 5 mg daily. Two 24 h ABPM measurements were performed, at baseline and the day of the first intake of minoxidil tablet. No significant differences were found in the 24 h mean SBP, DBP or HR between the two measurements. A slight reduction in SBP and DBP was observed in the first 2 h after taking minoxidil, normalizing to basal values after 4 h [31]. These time intervals are consistent with the pharmacokinetics of minoxidil, whose hypotensive effect begins 2 h after intake, and its plasmatic half-life is 4 h [1].

Imhof et al. published a study with 24 h ABPM measurement in 25 women treated with LDOM 0.625–2.5 mg, with the most frequent daily doses being 1.25 (52% of patients) and 2.5 mg (40%). In addition, LDOM was used in monotherapy and the patients’ mean age was 61 years, higher than in other studies. Two measurements were performed at baseline and after 4 months of treatment, finding a slight reduction in SBP (−2.8 mmHg) and DBP (−1.4 mmHg), along with an increase in HR (+4.4 bpm). Only one patient discontinued treatment due to edema in the lower limbs [32].

A recent study by Müller-Ramos et al. prospectively evaluated BP and HR in hypertensive patients treated with LDOM. Among 58 adults, there were no significant changes in BP and HR after 30 days of treatment, except for a modest decrease in DBP in those with elevated baseline readings [35].

### 4.2. Postural Hypotension, Orthostatism or Dizziness

Several studies have reported a low frequency of postural hypotension or orthostatism with LDOM. This AE is often described by patients as dizziness or lightheadedness, but clinical hypotension does not occur in most cases [2,4]. Even in cases with clinical hypotension, it is usually a transient AE with complete recovery [31]. In a study with 24 h ABPM, a 28-year-old patient developed an episode of dizziness (without syncope) after the first intake of minoxidil 5 mg, which was recorded in the ABPM device. His BP began to drop after 20 min of intake, reaching 91/49 mmHg after 90 min. After 3 h, his BP had normalized, with complete recovery of the patient [31].

Dizziness occurs in approximately 1–1.7% of patients and usually appears in the first week of treatment. It is more common in women, and may occur with relatively low doses of LDOM (mean 0.97 mg in women) [2,10,36]. Caution should be taken in patients with a personal history of orthostatic hypotension or syncope and, ideally, in those receiving treatment with calcium channel blockers [2,37]. However, in a study in hypertensive patients, no higher frequency of postural hypotension was found in patients taking this drug. It was observed that hypertensive patients treated with LDOM may present orthostatism more frequently than the general population, especially those taking three or more antihypertensive drugs or doxazosin [36].

Regarding the management of this AE, it is recommended to take the minoxidil tablet at bedtime, to avoid standing up too quickly and to increase fluid intake. Some authors also recommend taking licorice gum [9] or sodium chloride 50 mg daily [19], although the latter could potentially worsen or favor the appearance of edema. In hypertensive patients, they may be advised to self-monitor BP during the first days of treatment, in addition to taking minoxidil separately from other antihypertensive drugs [36,38].

When these measures are not beneficial, most cases are resolved by reducing the dose of LDOM. However, sometimes, it is necessary to discontinue LDOM due to dizziness, which is one of the most frequent causes of suspension [2,9].

## 5. Fluid Retention, Edema

Oral minoxidil may favor peripheral edema due to hydrosaline retention. The main mechanism seems to be the activation of the renin–angiotensin axis, which would increase the production of aldosterone [39]. Recently, it has also been suggested that minoxidil, as well as other drugs that act by opening potassium channels, may alter lymphatic smooth muscle contractions, thereby reducing lymphatic drainage and favoring the appearance of peripheral edema [40].

### 5.1. Frequency and Risk Factors

Fluid retention is one of the most frequent systemic AEs of LDOM, occurring in 1.3–10% of patients, according to various series. It is a delayed AE that appears between 1 and 3 months after initiation of LDOM, more frequently at 2 months [2,4,8].

It is typically more common in women, and in situations that favor fluid retention such as hot weather, obesity or prolonged standing or sitting [8,9,10]. In addition, there may be a potentially increased risk in patients taking calcium channel blockers or other sodium retainers, such as NSAIDs [37].

Similar to hypertrichosis, fluid retention has shown to be a dose-dependent AE [9,10,37]. A meta-analysis found that the dose of 1 mg appears to be a relevant threshold, as doses higher than 1 mg increased the risk of developing leg edema compared to doses lower than 1 mg [10]. This is consistent with Vaño-Galvan’s study, in which the mean dose to produce edema was 1.97 mg [2].

Additionally, a recent article by Salas et al. found a positive dose–weight relationship (mg/kg/day) between LDOM and the risk of edema [41].

### 5.2. Affected Areas

In most patients, fluid retention presents as bilateral leg edema in the pretibial or malleolar regions. Facial edema has been described in 0.3–1% of cases, and may be alarming for the patient [2,4]. It usually presents as bilateral periorbital or eyelid edema. Generally, it appears upon awakening in the morning, and typically resolves spontaneously during the day [42]. However, it may be persistent in some cases, requiring LDOM withdrawal.

Finally, generalized edema is rare and has been described in patients with severe AEs or associated with compounding errors in the dose [43,44,45].

### 5.3. Severity and Management

Leg edema is usually mild and self-limited within a few weeks or months, without the need for discontinue LDOM [2,8,41]. In these cases, restricting salt intake may be helpful. The recommended daily intake for healthy adults in USA is ≤2300 mg/day of sodium (equivalent to 1 teaspoon of table salt) [46]. However, other guidelines, such as the American Heart Association, set 1500 mg/day of sodium as the recommended upper limit [47]. In more persistent or concerning cases, a diuretic may be added. Loop diuretics, such as furosemide, used to be the most recommended group when minoxidil was used for hypertension [1,37]. However, spironolactone is an interesting option in women as it can also improve the AGA, even at low doses [2,9,48]. Its dual mechanism of action, with diuretic and anti-androgen effects, makes it an optimal drug for this situation. In other patients, it will be necessary to reduce the dose of LDOM, with resolution of the edema in most of them. Approximately 0.3% of patients require discontinuation of LDOM. In some cases, the drug has subsequently been reintroduced at a lower dose, with no recurrence of the AE [9,42].

## 6. Tachycardia, Arrhythmias and ECG Abnormalities

Palpitations are a very common AE with minoxidil at antihypertensive doses, which is mainly due to a reflex tachycardia or tendency to atrial arrhythmia produced by activation of the sympathetic nervous system (secondary to vasodilation) [1,37,39]. However, it appears to be a rare AE with LDOM, reported in 0.9–4% of patients [2,4,10]. In addition, among all the studies that measured HR in patients treated with LDOM, only one of them found a significant increase of 5 bpm at 24 weeks (Table 1) [28]. This is probably explained by the fact that tachycardia usually occurs in the first 3 days of treatment (most often in the first 24 h) and is usually mild and transient [2]. This increase in heart rate tends to normalize with chronic use of minoxidil [39]. In persistent or bothersome cases for the patient, it is possible to initiate a beta-blocker to manage tachycardia [2,37]. However, there is some consensus that it is more advisable to reduce the dose or discontinue LDOM in these cases or to request evaluation by a cardiologist [2,9,38].

Regarding other type of arrhythmias, Sanabria et al. evaluated the occurrence of supraventricular and ventricular extrasystoles using 24 h Holter in 34 men treated with LDOM 5 mg, showing no significant differences between baseline and 24 weeks [30]. In another study that included a subgroup of 10 patients with arrhythmia, no patient reported an increase or worsening of their arrhythmia [36]. However, in patients with a previous history of arrhythmia, a prior evaluation by a cardiologist is recommended [36].

Electrocardiogram (ECG) abnormalities may occur in up to 60% of patients with antihypertensive doses of minoxidil. They are usually transient and asymptomatic, and typically observed on heart repolarization [1]. In the case of LDOM, one study found alterations in the T wave in 20% of patients, mainly a non-ischemic inversion of the T wave in the V1 lead, with no clinical relevance for the patients [8]. Another study which performed 24 h Holter in patients treated with LDOM found no changes in the repolarization of the ECG [30].

## 7. Pericardial Effusion and Serious Adverse Effects

In the 1980s, during the first years of oral minoxidil use for hypertension, severe AEs such as myocardial infarction, pulmonary hypertension, left ventricular hypertrophy, pleural effusion, and pericardial effusion were reported [1,49]. The latter has been described in up to 3% of patients treated with minoxidil as an antihypertensive, leading to cardiac tamponade in some cases [1]. Unlike other cardiovascular AEs [12], pericardial effusion has been classically described as an idiosyncratic AE [9,50]. However, there is controversy in this regard as the mechanism appears to be due to a combination of dose-dependent neuro-hormonal changes (in the sympathetic nervous system and the renin–angiotensin axis) together with alterations in renal hemodynamic function, and a causal association with minoxidil could not be established [1,43]. In fact, most cases of pericardial effusion described with oral minoxidil have occurred at antihypertensive doses in patients with previous comorbidities or renal involvement due to different disorders, such as systemic lupus erythematosus, congestive heart failure, chronic kidney disease, or dialysis patients [1,43,49,51,52].

Regarding LDOM, only three case reports of pericardial effusion or pericarditis have been published to date (Table 2) [44,53,54]. However, only one of them specified whether minoxidil was a pharmaceutical compound or a commercially available drug. In many countries, especially in Europe and South America, oral minoxidil is not commercially available or it is only available in 10 mg tablets [55,56]. For this reason, or due to a shortage of 2.5 mg or 5 mg tablets [57], it is not uncommon for LDOM to be prescribed as a pharmaceutical compound, with the potential risk of dosage errors [58]. In this sense, a recent article by Moreno-Arrones et al. studied severe AEs of LDOM in the treatment of alopecia. This retrospective study described 12 cases of severe AEs between 2018 and 2020, accounting for 0.7% of all LDOM prescriptions during that period (approximately 1700). All cases were women, with a mean age of 46.5 years (range 25–73), with no personal history of cardiovascular disease or arterial hypertension. All patients had generalized edema (n = 6) or syncope (n = 6) or a combination of both, and two of them developed an ischemic stroke and a myocardial infarction, respectively. Interestingly, all of them were taking compounded minoxidil for treatment of AGA. The capsules were analyzed by a pharmacological laboratory, finding that most of them contained a real dose between 10 and 100 times higher than the one prescribed by the dermatologist. Specifically, the patient who had a stroke was taking a dose of 1000 mg instead of 1 mg. In addition, the majority of patients developed symptoms after the first intake of minoxidil, which supports the dose-dependent mechanism of cardiovascular AEs [45].

A recent study assessed the presence of pericardial effusion in patients treated with LDOM. A non-diagnostic, transthoracic ultrasound screening was performed by two board-certified cardiologists in 100 consecutive patients treated for alopecia. From them, 51 patients were on treatment with LDOM 2.5 or 1.25 mg (mostly women, mean age 53.7 years). Trivial effusions were detected at a similar rate between LDOM and control patients (35% of patients in each group). Small, asymptomatic pericardial effusions were observed in 5.8% of LDOM-treated patients and 6% in the control group, with no significant differences in the frequency between the two groups [59].

## 8. Other Adverse Effects

One notable AE of LDOM is a temporary increase in hair shedding shortly after starting treatment. Commonly known as “dread shed”, this phenomenon typically begins 2–4 weeks after initiating therapy and lasts about 3–6 weeks [60]. It is believed to occur due to a minoxidil-induced shortening of the hair’s telogen phase, leading to the accelerated shedding of hairs that would have naturally fallen out over the following weeks or months. It has been described in 16–32% of patients, although its frequency is thought to be underestimated [4,7,19]. While this shedding is usually temporary and often followed by improvements in hair density and thickness, it can be highly distressing for patients, particularly those already experiencing hair loss. A suggested approach to manage this AE is to overlap the use of topical minoxidil, if the patient is already using it, with oral minoxidil when starting LDOM therapy. However, a recent study by Nohria et al. suggests that this method does not mitigate the development of hair shedding [60].

Headache is described in up to 7% of patients using topical minoxidil [61,62] and it is usually assumed to be linked to the vasodilator effect of minoxidil. This AE has been increasingly reported with LDOM. Vaño-Galvan et al. described it in 0.4% of patients, while Sanabria et al., in a study conducted through an active survey, reported it in up to 9% of patients. It usually appears between 15 and 20 days of treatment, and improves with common analgesics, although it can sometimes be a reason for adjustment or suspension of LDOM [2,4]. Interestingly, a recent article by Desai et al. assessed the influence of several concomitant medications in 71 patients treated with LDOM for alopecia. They found that NSAIDs and triptans, commonly prescribed drugs for headache, do not negatively impact the effectiveness and tolerability of LDOM [63].

Other reported AEs include sleep disturbances, such as insomnia or nightmares [2,4]; increased appetite or weight gain (typically linked to fluid retention) [2,4]; nausea [22]; urticaria [19,22]; onset or worsening of acne [22]; dry mouth [4]; general malaise [36]; and exacerbation of alcohol hangover symptoms [64]. As discussed before, severe AEs are extremely rare with LDOM, and it is thought that they may be related to dosage errors or variability in compounding [45].

## 9. Summary and Future Directions

LDOM may be used as a first-line treatment for androgenetic alopecia in both men and women, supported by the existing literature and some academic guidelines [6,38]. Prior to starting LDOM, patients should be informed about its off-label use. In addition, a thorough patient history should be considered, particularly regarding cardiovascular conditions, peripheral edema, and blood pressure fluctuations. The patients should also be informed about potential AEs, such as hypertrichosis, postural hypotension/dizziness, fluid retention, tachycardia or headache.

For female patients, treatment can be started at 0.5 mg or 0.625 mg daily, increasing every three months if necessary, up to a maximum of 2.5 mg/day [6,38]. In male patients, treatment may start at 2.5 mg daily, with dose escalations every three months up to a maximum of 5 mg/day. However, some studies suggest that men may start with 5 mg daily with good tolerability, and even increase to higher doses (7.5 mg) [6,34].

In the general population, no routine complementary tests are required prior or during the follow-up treatment. The most common AE is hypertrichosis, which is typically mild and not concerning. Cardiovascular systemic AEs, such as edema, dizziness or tachycardia, are less common and generally well tolerated, while severe AEs, such as pericardial effusion, are extremely rare at low doses. Proper recognition and management of these AE by clinicians are essential to maximize the treatment’s benefits while minimizing risks (Table 3).

However, particular considerations should be taken in some special situations. Oral minoxidil is contraindicated in patients with pheochromocytoma or with allergy to any of the components of minoxidil tablet [1]. LDOM is not recommended in patients with a history of recent myocardial infarction, heart failure with left ventricular dysfunction, severe valve disease or advanced chronic kidney disease [36]. In these cases, the risk–benefit balance should be assessed and the treatment should be consulted and agreed upon with the appropriate physician (cardiologist, nephrologist).

In patients with an increased risk of AEs, such as patients with personal history of hypertension, arrhythmia, postural hypotension, peripheral edema or pericardial disease, LDOM should be used with caution. In addition to the aforementioned general measures to prevent systemic AEs, it could be reasonable to start with minimum doses and up-titrate slowly according to tolerability [6,36,38].

In conclusion, low-dose oral minoxidil is a valuable treatment for various types of hair loss, with a favorable safety and effectiveness profile. Future research should focus on long-term safety evaluation, especially in patients with pre-existing cardiovascular conditions. Further large-scale studies are needed to stablish standardized guidelines. Additionally, investigations on strategies to minimize AEs would be very valuable to further enhance the safety of this treatment, such as potential biomarkers to predict AEs, combination therapies to mitigate AEs, or novel formulations of minoxidil to reduce systemic exposure.

## Figures and Tables

**Table 1:** Summary of studies assessing blood pressure variation in patients treated with LDOM.

Study	Design	Results
Ong et al., 2024 [29]	n = 151 (89 women) LDOM 0.625–5 mg Retrospective Single measurement at baseline and with LDOM (mean 17 weeks)	- No significant variation in SBP or DBP - Only a significant slight reduction in DBP was found in men aged 35–49 years (81 → 78 mmHg)
Imhof et al., 2023 [32]	n = 25 (women) LDOM 0.625–2.5 mg Retrospective ABPM 24 h baseline and at 16 weeks	- Slight reduction in SBP (−2.8 mmHg) and DBP (−1.4 mmHg) - HR increase (+4.4 bpm) - Does not specify level of statistical significance
Jimenez-Cauhe et al., 2023 [31]	n = 10 (men), LDOM 5 mg Before-After Study ABPM 24 h baseline and the day of the first intake	- No significant differences in mean SBP, DBP or HR in 24 h ABPM - Slight and non-significant reduction in DBP and DBP in the first 2 h after taking LDOM
Sanabria et al., 2022 [30]	n = 34 (men), LDOM 5 mg Prospective ABPM 24 h at baseline and at 24 weeks	- **Significant slight reduction** in SBP (−3 mmHg; 125 → 122) and DBP (−2 mmHg; 76 → 74). - Non-significant increase in HR (+2.4 bpm; 72.5 → 74.9)
Panchaprateep et al., 2020 [8]	n = 30 (men), LDOM 5 mg Retrospective Single baseline measurement, 1 h after first dose and at 24 weeks	- 1 h after the first dose: non-significant variation in SBP (−2.5 mmHg), DBP (+0.5 mmHg) and HR (−4 bpm) - 24 weeks: non-significant reduction in SBP (−3.9 mmHg) and DBP (−1.1 mmHg). Non-significant increase in HR (+0.8 bpm)
Ramos et al., 2019 [28]	n = 30 (female), LDOM 1 mg RCT (vs. topical minoxidil) Single measurement at baseline and at 24 weeks	- Non-significant reduction in MAP (−2 mmHg; 93 → 91). No differences compared to the control group - **Significant increase** in HR (72 → 77 bpm), with differences with the control group

Abbreviations: ABPM: ambulatory blood pressure monitoring; DBP: diastolic blood pressure; HR: heart rate; LDOM: low-dose oral minoxidil; MAP: mean arterial pressure; mmHg: millimeters of mercury; RCT: randomized clinical trial; SBP: systolic blood pressure.

**Table 2:** Case reports of pericardial disease in patients treated with LDOM.

Article	Patient Characteristics	Minoxidil Treatment	Clinical Presentation	Intervention
Dlova et al., 2022 [44] South Africa	Female, 40 years old No prior comorbidities	0.25 mg for 3 weeks Not specified if it was a pharmaceutical compounding	Generalized edema (extremities and face), pericardial effusion	Hospital admission LDOM suspension IV Furosemide
Trüeb et al., 2022 [54] Switzerland	“Young, Healthy Woman” Age not specified	1.25 mg for “few weeks” Not specified if it was a pharmaceutical compounding	Dyspnea, chest pain, orthostatism, leg edema, pericardial effusion.	Not specified
Bentivegna et al., 2022 [53] USA	Male, 52 years old High intensity sport Personal history of pericarditis and pericardial effusion 5 years earlier	2.5 mg for 2 weeks Commercially available drug	Peripheral edema and pericarditis, without pericardial effusion	LDOM suspension Oral colchicine for 3 months

Abbreviations: IV: intravenous; LDOM: low-dose oral minoxidil.

**Table 3:** Summary of the main systemic adverse effects of LDOM and their management.

Adverse Effect	Characteristics	Management
**Leg edema**	- 1.3–4% of patients - Appears at 2 months (45–90 days) - More common in women and hot weather	- Restriction of salt intake - Furosemide, spironolactone (women) - Reduce or discontinue LDOM
**Periorbital or facial edema**	- 0.3–1% of patients - Usually in the morning and self-resolving in minutes or hours	- Explain and calm the patient - Reduce or discontinue LDOM if persistent
**Dizziness, lightheadedness, postural hypotension**	- 1–1.7% of patients - Appears in the first week - More common in women and patients on antihypertensive treatment	- Take LDOM at night; increase water intake; take sodium chloride or licorice gum - Adjust antihypertensive - Reduce or discontinue LDOM
**Tachycardia**	- 0.9–4% of patients - Typically appears on the first day (1–3 days) - Usually mild and transient	If persistent: - Refer to cardiologist - Reduce or discontinue LDOM - Add beta-blockers
**Headache**	- 0.4–9% of patients - Appears at 15–20 days - Usually mild and transient	- Common analgesics - Reduce or discontinue LDOM if persistent
